# Another look at Emergency Department HIV screening in practice: no need to revise expectations

**DOI:** 10.1186/1742-6405-7-1

**Published:** 2010-01-05

**Authors:** Jeremy Brown, Manya Magnus, Maggie Czarnogosrki, Vanessa Lee

**Affiliations:** 1 Department of Emergency Medicine, The George Washington University Medical Center, Washington DC, USA; 2 Department of Epidemiology and Biostatistics, The George Washington University School of Public Health and Health Services, Washington DC, USA; 3 National Institute of Allergy & Infectious Diseases, Bethesda MD, USA

## Abstract

**Background:**

A recent study reported a lower than expected specificity and positive predictive value of the rapid oral HIV test in the setting of routine emergency department (ED) screening. These results appeared inconsistent with the findings in another urban Emergency Department during the same time period.

**Objective:**

To compare the specificity and positive predictive vale (PPV) of an oral rapid HIV test used in an ED screening program in Washington DC with that performed in the USHER clinical trial.

**Design:**

Period cross-sectional analysis of rapid oral HIV testing conducted in an ongoing HIV screening program emergency department patients.

**Setting:**

The George Washington University Emergency Department (Washington DC) from 7 February to 1 October 2007.

**Patients:**

1,560 adults seen in the ED for non-HIV-related presenting complaints, who participated in the HIV screening program.

**Intervention:**

Rapid HIV testing with the OraQuick *ADVANCE Rapid HIV-1/2 Antibody Test *(OraSure Technologies, Bethlehem, Pennsylvania). Patients with reactive rapid test results were offered Western blot testing for confirmation.

**Measurements:**

Specificity and positive predictive value for the program were determined. Findings were compared to those found in the USHER trial.

**Results:**

Of 1,560 patients screened for HIV, 13 [0.8%, 95% CI 0.38% to 1.28%] had a reactive HIV screening test, and all were confirmed to be positive by Western Blot. The specificity was 100% (95% CI 99.6%-100%).

**Limitation:**

Since non-reactive tests were not confirmed, the test sensitivity cannot be determined.

**Conclusion:**

Review of our data conflict with findings from the USHER study surrounding false positive OraQuick HIV screening. Our data suggest that rapid HIV screening protocols implemented in EDs outside of the clinical trial paradigm perform effectively without an excess of false positive results. Compared with other screening tests, HIV rapid screening should remain an essential component of ED practice.

## Background

Since the Centers for Disease Control and Prevention (CDC) recommended routine opt-out HIV screening in emergency departments in 2006,[1] the George Washington Emergency Department has been conducting routine HIV testing among all patients that present for care[2]. This clinical program (rather than a research trial) is funded by the DC Office of HIV/AIDS, the CDC and unrestricted pharmaceutical grants. It operates in a real-world setting among a diverse population of patients seeking ED care.

In August 2008, the investigators for the Universal Screening for HIV infection in the Emergency Room (USHER) Trial published a report which described a larger than expected number of false positive tests [3]. This led the authors to suggest that expectations from rapid HIV testing in the emergency department should be revised. As evidence of the importance of this paper, the journal in which these results were published printed a one page patient summary [4]. The USHER Trial analyzed data from 849 patients between 2/7/07 and 10/1/07, and found, of those who allowed confirmatory Western Blot testing, that 26 of their 39 preliminary positive results were not confirmed positive; this revealed a false positive rate that exceeded expectations that were based on parameters listed within the OraQuick product insert. The purpose of this study was to examine the impact of a real-world, non-clinical trial ED HIV screening program during the same time span and to examine the prevalence of false positives.

## Methods

### Clinical protocol and test site

The George Washington University Hospital is an academic urban medical centre in the District of Columbia. The annual census in the emergency department is over 60,000. Of these, 53% are African-American and 33% are white. Unlike the USHER trial, in which participants were consented to participate in the study and then randomized to receive rapid HIV testing in the ED by a dedicated HIV counsellor or by a staff member at the ED, all GWU patients between the ages of 13 and 64 years presenting to the emergency department are eligible for an HIV screening test if they speak either English or Spanish. Patients who know they are HIV positive, who have been tested for HIV in the last three months, who have an altered mental status, or who require urgent medical intervention are excluded from screening. Screening was offered by trained additional staff and two screeners were assigned to periods of peak activity in the ED. Screeners were available from 8 am-midnight every day. The screening staff was made up of extensively trained undergraduate health sciences students.

Patients that were not critically ill, whether they were ambulatory patients or had arrived by ambulance were informed of the availability of a free HIV screening test at the point of triage. They were given written information about HIV disease, and were informed of the importance of HIV testing by the triage nurse. However the triage nurse did not ask the patient to whether or not they would accept an HIV test when offered to them. HIV screeners then approached the patients and offered them a rapid HIV screening test. Patients who accepted screening were tested with an oral swab using the OraQuick *ADVANCE *Rapid HIV-1/2 Antibody test (OraSure Technologies Inc, Bethlehem PA). Testing was performed in parallel to the provision of standard ED care. Results were available within 20-40 minutes and negative results were relayed to the patient by the screener. All patients with a negative screening test were given HIV risk reduction materials, and the results were noted on the ED record. All positive screening results were reviewed by a second screener and the ED attending physician. If there were consensus surrounding the positive result, the ED attending physician informed the patient of the test result as well as the preliminary nature of the screening result in a confidential area. Unlike the clinical trial setting found in the USHER study, laboratory investigation with HIV RNA PCR and CD4 counts were not available to further assess patients. A local testing algorithm was implemented to avert inconclusive and false positive results: patients who had a weakly positive test were screened a second time using whole blood. If the result were positive twice, it was recorded as a preliminary positive test result in the ED records. All patients with a preliminary positive test were offered a Western Blot confirmatory test. They were also linked to care with the hospital's Division of Infectious Diseases clinic where the results of the confirmatory Western Blot test were disclosed, and further care was arranged as needed. Data on age, gender, race, zip code of residence, acceptance or refusal of HIV testing, and the test results were collected for all patients by the screening personnel.

To explore the possibility of a cluster of inaccurate OraQuick test kits or other barriers to effective ED screening, we reviewed the outcomes of the tests from 7 February to 1 October 2007, the same time span reported in the USHER Trial paper [3]. This allows for comparison between two sites as well as two testing algorithms during the same time.

### Role of the funding sources

Since its inception, the GWU ED HIV testing program has been supported by the DC Department of Health, the Centers for Disease Control, and from unrestricted grants from Gilead Sciences. The decision about which test kit to use was made by the DC Dept of Health, which then provided the kits to the testing site. None of these organizations had any role in the study design and interpretation of the results, or in the decision to submit the manuscript for publication.

### Evaluation of the screening performance

The specificity of the screening test is defined as the proportion of screened patients who had a negative rapid test among non-HIV infected patients. The positive predictive value (PPV) is calculated as the proportion of patients with a preliminary reactive rapid test that are infected with HIV as confirmed by Western Blot. Sensitivity cannot be calculated given that the negative population seen in the GWU ED, as with the USHER trial, do not contribute specimens for confirmatory testing, and patients with a negative screening test were not retested and did not have confirmatory Western Blot or other HIV-related measures collected. Both are consistent with standard HIV screening practice. All 95% confidence intervals were calculated on the basis of the normal approximation of the binomial distribution [5]. In order to assess test parameters under a variety of assumptions regarding the proportion of both false negatives and false positives, a sensitivity analysis was performed. The scenarios looked at the resulting sensitivity, specificity, and PPV had there been 0, 1, and 28 (1.2%) false negatives, 0, 2 (0.95% overall HIV prevalence), and 4 (1.1% overall HIV prevalence) additional false positives among the negatives, ad the combination of each of these. Data were analyzed using Stata version 9.0SE (College Station, TX).

## Results

### Sample

**Figure 1:**
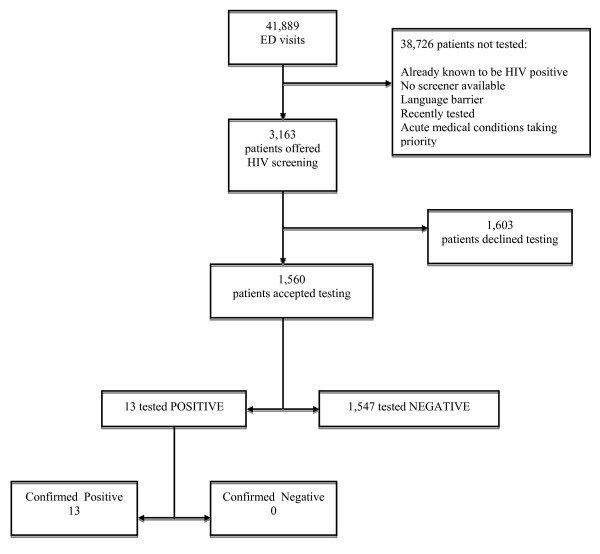
**Study flow**.

From 2/7/07 to 10/1/07, there were 41,889 visits to the ED. Of these, 3,163 patients were offered an HIV screening test, representing 7.5% of all ED patients seen over this period of. A total of 1,560 (49%) agreed to be tested and all received their results in the ED (Figure 1). Of those tested, 1,547 were negative [99.2%, 95% CI 98.7% to 99.6%] and 13 [0.8%, 95% CI 0.38% to 1.28%] were preliminary positive. There were no invalid tests. Patients with a reactive test were more likely to be male (*p *< 0.05) and black (*p *< 0.10), as shown in Table 1. Otherwise, the demographic characteristics between patients with a reactive and a non-reactive result were similar.

**Table 1:** Characteristics of Patients tested for HIV

Characteristic	Reactive test (n = 13) n(%)	Non reactive test (n = 1547) n(%)
**Mean age (SD), yrs**	34 (8)	35 (12.6)

Men, n (%)*	11 (84)	681 (44)

**Race n(%)**		

**White**	2 (15)	554 (36)

Black**	10 (77)	826 (53)

**Other**	1 (8)	167 (11)

**Ethnicity**		

**Hispanic**	1 (7)	64 (4)

**Non-Hispanic**	12 (93)	1483 (96)

*p < 0.05

**p < 0.10

### Test performance

All 13 patients (out of 1,560 tested) with a reactive test agreed to confirmatory testing, and all were confirmed positive by Western Blot. Therefore, the occult HIV prevalence rate was 0.8% during the study period [95% CI 0.38% to 1.28%]. Assuming that the nonreactive tests were truly negative, the test specificity was 100% (95% CI 99.8%-100%). Although the observed sensitivity and observed false negative rates could not be calculated because non-reactive tests were not confirmed with a Western Blot, a series of scenarios were developed to place bounds on what might be expected. While specificity remains constant in the face of false negatives, sensitivity with 0, 1, and 28 false negatives ranges from 100.0% to 31.7% at each extreme. The primary concern that observed negatives could have been false positives (or that the use of the enhanced testing algorithm to retest weak results could have obscured such a finding) was explored by looking at sensitivity and specificity for 0, 2, and 4 additional false positives (out of observed negatives). In this case, the sensitivity reduces to 86.7% under the assumption of 2 false positives and 1.2% false negatives. Specificity remains in the high 90% range throughout (Table 2).

**Table 2:** Sensitivity Analysis

		Assuming *X *of the negatives were actually false positives		
		
		0 (observed)	2 (0.95% prevalence of positives overall)	4 (1.1% prevalence of positives overall)
**False negatives**	**0**	Pr(+|A) 100% (95% CI 75.3%-100%)	Pr(+|A) 100% (95% CI 75.3%-100%)	Pr(+|A) 100% (95% CI 75.3%-100%)
		Pr(-|N) 100% (95% CI 99.8%-100%)	Pr(-|N) 99.9% (95% CI 99.5%-100%)	Pr(-|N) 99.7% (95% CI 99.3%- 99.9%)
		Pr(A|+) 100% (95% CI 75.3%-100%)	Pr(A|+) 86.7% (95% CI 59.5%-98.3%)	Pr(A|+) 76.5% (95% CI 50.1%-93.2%)
	
	**1**	Pr(+|A) 92.9% (95% CI 66.1%-99.8%)	Pr(+|A) 92.9% (95% CI 66.1%-99.8%)	Pr(+|A) 92.9%(95% CI 66.1%-99.8%)
		Pr(-|N) 100% (95% CI 99.8%-100%)	Pr(-|N) 99.9% (95% CI 99.5%-100%)	Pr(-|N) 99.7% (95% CI 99.3%-99.9%)
		Pr(A|+) 100% (95% CI 75.3%-100%)	Pr(A|+) 86.7% (95% CI 59.5%-98.3%)	Pr(A|+) 76.5% (95% CI 50.1%-93.2%)
	
	**28** **(1.2% false negatives)**	Pr(+|A) 31.7% (95% CI 18.1%-48.1%)	Pr(+|A) 86.7% (95% CI 59.5%-98.3%)	Pr(+|A) 24.3% (95% CI 11.8%-41.2%)
		Pr(-|N) 100% (95% CI 99.8%-100%)	Pr(-|N) 100% (95% CI 99.8%-100%)	Pr(-|N) 99.7% (95% CI 99.3%-99.9%)
		Pr(A|+) 100% (95% CI 75.3%-100%)	Pr(A|+) 100%(95% CI 75.3%-100%)	Pr(A|+) 69.2% (95% CI 38.6%-90.9%)

In order to expand the time period from that explored by USHER, we also combined our sample described here with our sample of patients previously described elsewhere [6]. From the resulting combined sample of 4,046 patients at GWU, the specificity is 99.9% and the PPV is 85% (Table 3).

**Table 3:** Oral rapid HIV test performance

Study	Total tested	Negative	Reactive (preliminary positive)	Lost to follow-up	True Positive	False Positive	95% CI for false positives	Specificity	PPV	Unconfirmed cases included as		
										
										HIV Negative Specificity	HIV Positive Specificity	HIV Negative PPV
**Walensky **[3]**BOSTON**	849	810	39	8	5	26	16-35	96.89%	16.13%	95.97%	96.89%	12.82%

**Brown** **DC**	1560	1547	13	0	13	0	0-4	100%	100%	100%	100%	100%

**Brown **[9] **DC**	2486	2460	26	13	9	4	1-8	99.84%	69.23%	99.31%	99.84%	40.91%

**DC** **Totals only**	4046	4007	39	13	22	4	1-8	99.90%	84.62%	99.58%	99.90%	56.41%

## Discussion

This study revealed that the oral rapid HIV screen performed as expected, and that the number of false positive tests was as predicted by the manufacturer. The earlier published report by Walensky of "a false-positive rate fifteen times greater than the anticipated specificity of 0.2%" was not supported by the evidence from our clinical program involving almost twice as many patients. Under extreme scenarios of false positives among negatives as false negatives alone and combined, the sensitivity remained at an acceptable level for a rapid screening test administered in an ED setting, comparable with other screening tests used on a population level.

There have been a number of reports addressing a higher than expected false positive rate. In 2006 Delaney reported the performance of the rapid oral HIV test in four CDC studies and reported that there was a small cluster of 16 false positive tests in one site [7]. This site had a specificity of 99% which was lower than in the other three studies, which reported a specificity of 99.6-99.8%. Jafa *et al *described an increase in the false-positive rate that occurred in Minnesota in 2006 [8]. The field investigation could not identify a cause for the increase. More recently, a report in 2008 described two episodes of an unexpected increase in the false positive rate in New York City [9]. It remains unclear if these are randomly occurring clusters, user-end failure, test kit failure, or other phenomena. Broader prospective studies of the overall accuracy of the test are needed in real-world settings, however, in order to fully understand these clusters.

### Limitations

The first explanation of these differences is that the two sites use different screening protocols. At The George Washington University ED, if the positive test line is only weakly visible, the screeners are directed to ask the patient for a sample of blood on which the test is run a second time. If this second sample is positive (even if weakly so) the test is considered to be reactive. If this second sample is negative, the test is recorded as negative. All reactive samples are considered preliminary and require confirmation with a Western Blot. This algorithm has been described elsewhere as likely to reduce the number of false-positive cases [7,10]. It is possible that the reactive results represent a subset of those that would have been reported had we used the algorithm in the product insert. However, since the number of weakly reactive oral tests requiring a second rapid test with whole blood is not recorded in our database, we cannot determine the size of the total subset of weakly reactive test. For this reason it is important to use caution when comparing reports of testing outcomes between sites, even if the product being used is identical. Our sensitivity analysis attempts to measure the impact of this testing algorithm and reveals that false negatives and false positives would not have eliminated the utility of the rapid HIV test except in the most extreme cases.

Although the number of patients we report is considerably larger than the USHER Trial report, the total number of reactive results (13) was fewer than those reported in the USHER Trial (39). Confidence intervals for the frequency of false positives are wide for both settings. In GW there were no false positives, with a 95% CI of 0-4 cases. In the USHER Trial, there were 26 false positives, with a 95% CI of 16-24 cases.

What are we to make of these findings? We believe that they are best understood when compared to other widely accepted screening tests that are performed. For example the PPV of the newborn screen for hypothyroidism is only 1.8%, and there are about 50 false positive results for every true-positive result identified through the US newborn screening program [11]. The false positive rate for a 40-year-old woman having her first mammogram is 7-10% [12]. The PPV of first-screening mammography (number of breast cancers detected per abnormal examination) increases with age from 3% for those aged 30 to 39 years up to 19% for those aged 70 years or older [13]. The USHER data demonstrated a lower than expected specificity, but one that is nevertheless far higher than specificity of these other accepted screening tests.

However, ED HIV screening is still in its infancy, and many hospitals are still undecided about whether or not to implement the CDC recommendation and screen ED patients for HIV [14]. The prior published findings of the USHER group are likely to dissuade such sites, given their number of false positive screening tests (26/31) and their low predictive values. Improved quality assurance and immediate verification with a second rapid test may have decreased the numbers of false positives in the USHER Trial. Our protocol, and others that have more recently been suggested [10] may be incorporated into routine ED HIV testing with the aim of decreasing the false positive rate. For this reason we believe that it is critical that the test characteristics of rapid HIV screening from other venues be reported and reviewed.

## Conclusions

We remain enthusiastic about routine ED HIV screening. Our analysis of the test characteristics demonstrates that the test outperforms many other commonly used screening tests. Given the potential for improved individual-level and population-level health as well as HIV prevention through identification and treatment of HIV-positive persons, there remains a great need for rapid testing in EDs. The real challenge is not whether or not the test performs as intended, but rather how to increase the numbers of patients who agree to be screened, which remains only 50-60% of all those eligible [15].

## Competing interests

JB has received lecture fees from OraSure.

## Authors' contributions

JB conceived the study and wrote the manuscript. MM performed the data analysis and reviewed the manuscript. MC performed patient follow up and reviewed the manuscript. VL performed data abstraction and manuscript review.

All authors have read and approved the final manuscript.
